# Trachomatous Trichiasis and its Management in Endemic Countries

**DOI:** 10.1016/j.survophthal.2011.08.002

**Published:** 2012-03

**Authors:** Saul N. Rajak, J. Richard O. Collin, Matthew J. Burton

**Affiliations:** 1 International Centre for Eye Health, London School of Hygiene and Tropical Medicine, London, UK; 2 Moorfields Eye Hospital, London, UK; 3 NIHR Biomedical Research Centre, Moorfields Eye Hospital and UCL Institute of Ophthalmology and UCL Partners AHSC, London, UK

**Keywords:** Entropion, eyelash, eyelid, surgery, trachoma, trichiasis

## Abstract

Trichiasis is the sight-threatening consequence of conjunctival scarring in trachoma, the most common infectious cause of blindness worldwide. Trachomatous trichiasis is the result of multiple infections from childhood with *Chlamydia trachomatis*, which causes recurrent chronic inflammation in the tarsal conjunctiva. This produces conjunctival scarring, entropion, trichiasis, and ultimately blinding corneal opacification. The disease causes painful, usually irreversible sight loss. Over eight million people have trachomatous trichiasis, mostly those living in poor rural communities in 57 endemic countries. The global cost is estimated at US$ 5.3 billion. The WHO recommends surgery as part of the SAFE strategy for controlling the disease.We examine the principles of clinical management, treatment options, and the challenging issues of providing the quantity and quality of surgery that is needed in resource-poor settings.

## I Introduction

Trachoma is the leading infectious cause of blindness worldwide^108^—a major ophthalmic public health problem in some of the world's poorest regions. Recurrent *Chlamydia trachomatis* infection in childhood results in entropion and trichiasis many years later. This may cause painful and (usually) irreversible blindness.

The World Health Organization (WHO) is leading a global campaign to eliminate blinding trachoma by the year 2020 (GET2020) through the implementation of the SAFE Strategy: **S**urgery for trichiasis, **A**ntibiotic distribution to control chlamydial infection, **F**acial cleanliness, and **E**nvironmental improvements to reduce its transmission.^145^ Trichiasis is the major risk factor for corneal opacification,^30^ and thus treating trichiasis is central to preventing visual loss. There are many challenges, however, in the effective management of trichiasis. The backlog of untreated cases remains high, reported surgical outcomes are often disappointing, endemic communities are frequently inaccessible, and questions remain over the optimal procedure, surgical training, surgical quality, and productivity. We examine the principles of clinical management, treatment options, and the challenging issues of providing the quantity and quality of surgery that is needed in resource poor settings.

## II Global Distribution and Burden

In 2009 WHO estimated that approximately 41 million people have active trachoma.^88^ This represents a marked decrease from 81 million in 2003, probably reflecting the success of the “AFE” components of control programs, more accurate disease estimates, and economic development in some trachoma endemic areas.^146^ Over the same period, however, there has been a modest increase in the backlog of unoperated trachomatous trichiasis (TT) from 7.6 million to 8.2 million cases, despite expanding trichiasis surgery services in many endemic countries. It may take many years before the control of *C. trachomatis* infection in endemic communities translates into a reduction in the incidence of new trichiasis cases.

Trachoma is currently endemic in 57 countries, most of which are in sub-Saharan Africa and Asia.^88^ The highest disease prevalence estimates come from countries in the Sahel belt and East Africa. Areas of Ethiopia and Sudan report active disease in 60% of children and trichiasis in 10% of adults.^53,83^ The economic impact of blinding trachoma on individuals and communities is high, particularly as affected communities are already poor. The most recent published estimate of the economic cost of visual loss from trachoma, which is thought to affect over 2 million people, is US$ 5.3 billion per year and significantly more if other debilitating symptoms of the disease, such as photophobia and pain, are taken into account.^56^

## III Pathophysiology and Natural History of Trachomatous Trichiasis

**Fig. 1:**
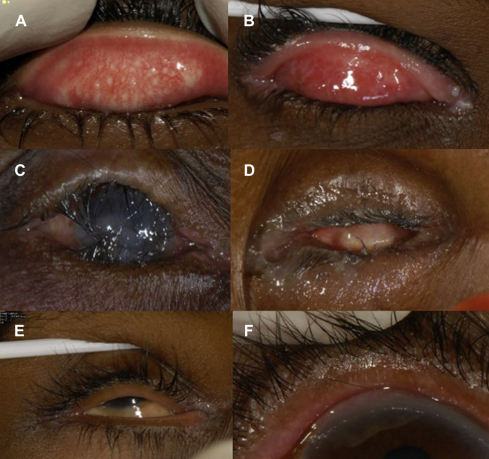
The clinical signs of trachoma. *A*: Active trachoma with both follicles and intense inflammation. *B*: Trachomatous conjunctival scarring. *C*: Entropion trichiasis and corneal opacity. *D*: Phthisis. *E*: Misdirected lashes. *F*: Metaplastic lashes.

The disease process begins in early childhood with recurrent *Chlamydia trachomatis* (Serovars A, B, B1, and C) infection of the conjunctival epithelium, which provokes a follicular conjunctivitis, known as active trachoma (Fig. 1A). This is characterized by an inflammatory cell infiltrate and a pro-inflammatory cytokine response.^27,50,95^ In endemic communities children are repeatedly infected, and this causes chronic inflammation.^137^ This leads to conjunctival scarring (Fig. 1B), cicatricial entropion, and trichiasis (Fig. 1C). Although episodes of *C. trachomatis* infection become shorter and less severe as individuals get older, inflammation still occurs, and scarring worsens over many decades.^7,26,31^ Eventually, the abrading lashes or secondary microbial keratitis cause corneal opacification (Fig. 1C). Severe microbial keratitis can ultimately result in phthisis (Fig. 1D). The visual loss is both painful and irreversible, as keratoplasty is rarely available and is at high risk for failure in trachoma-endemic countries.^151^

## IV Clinical Assessment

The vast majority of individuals with TT live in trachoma-endemic countries. However, with migration, TT cases occasionally present to ophthalmologists elsewhere. Patients are usually symptomatic. They may report eyelashes touching their eye or have less-specific symptoms such as foreign body sensation, tearing, pain, or photophobia. Many patients self epilate and should be asked about this to help gauge the severity of the trichiasis, although in some settings they may be reticent about admitting to this. Other improvised or traditional treatments, such as cutting eyelashes or using hot ash to burn away lashes, may have been tried. It is important to ask about previous lid surgery, including operations performed by traditional healers, as repeat surgery is usually more difficult. A family history can be informative: *C. trachomatis* infection and the disease it causes tend to cluster, with some families more severely affected than others. An individual's risk of developing scarring is a complex interaction between the lifetime exposure to *C. trachomatis* infection and the human immuno-fibrogenic responses.

In assessing patients with TT for treatment, several features need to be evaluated: lid and lash position, the tarsal conjunctiva, and the cornea. WHO has developed several systems for the clinical grading of trachoma. Two of these are currently in use: the detailed “FPC system”, which is primarily a research tool (Table 1) and the Simplified WHO Trachoma Grading System (Table 2), which is in routine use in control programs.^44,120^ The examination can be performed with a pair of loupes and a handlight. Visual acuity should be measured.

### A Lid and Lash Position

Lashes may come into contact with the globe for several reasons that may coexist in the same eyelid: (1) entropion, (2) misdirected lashes (abnormal direction, but originating from follicles that are in the lash line; Fig. 1E), and (3) metaplastic lashes (originating from follicles that are in an abnormal location; Fig. 1F).^10,103a^ The number and location of lashes touching the globe should be noted to gauge the severity. A few temporal lashes touching the conjunctiva are less problematic than central lashes in constant contact with the cornea. TT may occur with or without frank entropion. There is no standardized entropion grading system; it is useful to note the extent and degree of any entropion by looking for inward rotation of the lid margin in the primary position, however. In established entropion there is often “rounding” of the normally sharp edge where the tarsal conjunctival joins the lid margin. Lagophthalmos can occur in severe disease or as a result of previous lid surgery.^77^ Although TT occurs primarily in the upper lid, we have observed some people have lower lid TT.^102a^ For research purposes trichiasis has sometimes been subdivided, based on severity, into minor TT (five or fewer eyelashes touching the globe) and major TT (six or more eyelashes touching the globe).^105^

### B Tarsal Conjunctiva

The upper eyelid should be everted and examined for signs of trachoma. Adults rarely have active follicular conjunctivitis; however, eyes with TT frequently have inflammation of the tarsal conjunctiva. This could be caused by infection (chlamydial and non-chlamydial), mechanical irritation, or an immune-mediated response.^30^ Conjunctival scarring ranges from a few fine white lines to extensive dense scar tissue. Sometimes an Arlt's line is seen—a thick, dense, horizontal band of scarring. Entropion brings squamous epithelium from anterior to the gray line into a new environment, where factors, perhaps in the tears, cause it to take on a conjunctival phenotype. This gives the appearance of an anterior movement of the muco-cutaneous junction, normally posterior to the meibomian gland orifices, described as “conjunctivalization of the lid margin.”^77^ In individuals with more severe conjunctival scarring there may be squamous metaplasia secondary to chronic inflammation and dryness of the ocular surface.

### C Cornea

The cornea may develop opacification from direct trauma by the TT or from secondary bacterial infection. Fibrovascular pannus usually affects the upper third of the cornea, although this tends to be more prominent in children with active trachoma. Herbert's pits are small depressions, often pigmented, at the superior limbus and are the residua of limbal follicles. In severe cases the globe may become phthisical. Non-trachomatous causes of corneal opacity, such as microbial keratitis, trauma, vitamin A deficiency, and congenital measles, are also relatively common in trachoma endemic communities.

### D Differential Diagnosis

Cicatrizing conjunctivitis can develop in a wide variety of conditions. In some of these, the scarring may be severe enough to distort the eyelids, leading to entropion and trichiasis. Infectious causes include endemic trachoma (*C. trachomatis*), adenovirus, and *Corynebacterium diphtheria*. Several autoimmune conditions cause conjunctival scarring: mucus membrane pemphigoid, Stevens-Johnson syndrome, graft-versus-host disease, atopic keratoconjunctivitis, and linear IgA disease. Conjunctival scarring can also develop in ocular rosacea, sarcoidosis, following chemical injuries, and with specific drugs (practolol, topical adrenaline). Metaplasia and misdirection of the eyelashes in the absence of entropion can develop in inflammatory conditions that affect the lid margin (e.g., blepharitis and ocular rosacea).

## V Anatomy of the Upper Eyelid

**Fig. 2:**
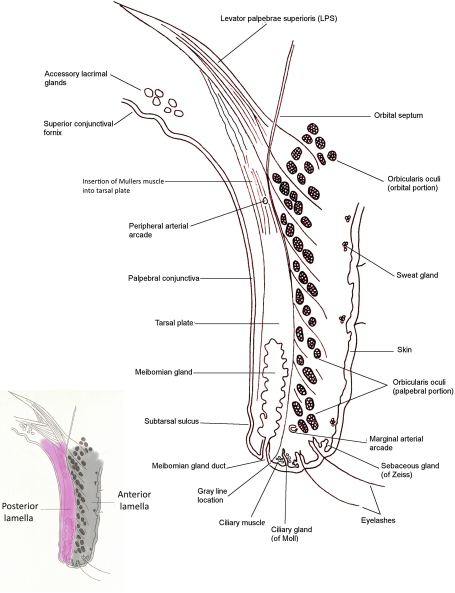
Cross-section of the upper eyelid.

The upper eyelid is formed of the anterior and posterior lamellae, separated superiorly by the orbital septum (Fig. 2). The anterior lamella consists of skin, subcutaneous tissue, the orbicularis oculi muscle fibers, and the insertion slips of the levator aponeurosis. The posterior lamella is formed of the tarsal plate containing the meibomian glands, sub-epithelial conjunctival stroma, Müller's muscle (continuous with levator palpebrae superioris), and the conjunctival epithelium. The upper eyelid receives its blood supply through an anastomosis of the medial and lateral palpebral arteries, branches of the ophthalmic artery. The palpebral arteries form the marginal and peripheral arterial arches, which run the length of the lid. Venous blood drains medially into the ophthalmic and angular veins and laterally into the superficial temporal vein. The lymphatic drainage from the lateral two-thirds of the upper lid is to the superficial parotid nodes, and the medial third drains to the submandibular node. The upper lid receives its sensory innervation from the ophthalmic division of the trigeminal nerve via the infratrochlear, supratrochlear, supraorbital, and lacrimal nerves. Orbicularis oculi is innervated by temporal and zygomatic branches of the facial nerve, and the smooth muscle is supplied by sympathetic nerve fibres from the superior cervical ganglion. Superiorly the orbital septum demarcates the upper end of the anterior lamella from the pre-aponeurotic fat pad.

## VI A Historical Perspective on Trichiasis Treatment

Trachoma was endemic in the ancient world. The earliest known written references are found in The Ebers Papyrus from Egypt (ca. 1600 BCE) which advises treatment with myrrh, lizard, or bat's blood or simply pulling out the lashes for trichiasis; and in the writings of Susruta in India (ca. 1000–500 BCE), which recommends cautery, lid incision, and everting sutures for entropion and trichiasis.^25,34,69^ Forceps thought to have been used to remove eyelashes have been found in many Egyptian tombs. The Hippocratic corpus (5th–4th century BCE) contains the first known use of the word *trichosis* (abnormal hair growth of the eye) and gives what is probably the earliest surviving description of a surgical treatment where it instructs that *trichosis* of the eye should be treated by:

… pass [ing] a thread through the eye of a needle; then piercing through along the angle of the upper extension of the eye-lid in a downward direction, draw the needle through; do the same again below this. Now pulling the threads tight, stitch them together, and keep them bound fast until the fall off. If this suffices, fine; if it fails, do the same again.^41,68^

Surgery for trichiasis was said to be one of the more common procedures in the Roman Empire.^74^ The operation involved eyelash epilation and lash follicle cautery with a hot iron, which has parallels to modern lash-root cautery procedures. Although the disease had evidently been present and described for millennia, the first surviving use of the word trachoma, from *trachys* [rough], is in the *Materia Medica* of Dioscorides Pedanius, written in the 1st century CE.^46^ Soon after Galen (129–200 CE), classified trachoma into five stages that are similar to those that we use today and included sycosis (scarred) and tylosis (trichiasis).^58^ In the 7th century CE, Paul of Aegina wrote a major medical compendium drawing on the medical knowledge of Hellenistic Alexandria and the Roman physicians. He described the treatment of trichiasis by removing a piece of eyelid skin by clamping a fold of skin between two pieces of reed. This technique produces a degree of lid eversion and was still used by traditional healers in Myanmar in the 1970s.^140^ Trichiasis treatment remained essentially unchanged from ancient times until the 19th century, when new oculoplastic procedures started to be developed for entropion.^3,117^

## VII Treatments for Trichiasis and Entropion

During the course of the last century many different procedures for the treatment of TT have been described. Trachoma-control programs in endemic countries routinely use some of these; others are rarely performed except by oculoplastic specialists. The variety of options suggests that, on the one hand, the “perfect” solution does not exist and that the treatment should ideally be tailored to the individual. On the other hand, the high prevalence of TT and the limited surgical services in most endemic countries demands a simple, quick procedure that can be taught to and carried out by non-ophthalmologists with the most basic equipment in the most basic settings. Treatment options broadly divide into those that only treat the lashes and those that also correct the underlying anatomical eyelid abnormality.

### A Lash Treatments

#### 1 Epilation

Individuals with trichiasis often pull out their own eyelashes, usually with locally made forceps, but sometimes with fingers.^23,135^ Indeed, it is not uncommon to meet patients who have removed all of their lashes. In some settings patients who do not have access to forceps use hot coals to burn away lashes or scissors to cut them. There is limited data on the effectiveness of epilation in preventing blindness. In one large cross-sectional analysis, epilation was common and associated with a significantly lower risk of corneal opacification in eyes with “moderate” or “severe” entropion, but no difference was found for eyes with only “mild” entropion.^135^ However, corneal opacification occurred in less than 10% of patients in this study with “mild” entropion.

It has been suggested that poorly conducted epilation leaves broken, short, sharp lash stubs that do more damage than the original long lashes,^44,107^ but there is no published data to support this view. Patients with trichiasis tend to avoid gaze positions that lead to contact between sharp lashes and the cornea. Additionally, if the lash is not broken during epilation, the re-growing lash does not appear to be sharper or more abrasive than the one it replaces.

There is a consensus that surgery is indicated for moderate to severe trichiasis, especially if it threatens the cornea. In contrast, opinion and practice are divided on the role of epilation in the management of mild TT. There have been no trials comparing repeated epilation to surgery for mild disease. Many ophthalmologists and some national trachoma control programs epilate patients with only a few lashes touching the eye that do not threaten the cornea.^22^ The majority of national trachoma control programs follow the current WHO guideline to offer tarsal rotation surgery to all patients with TT irrespective of number of trichiatic lashes, their location, or the severity of entropion.^148^ The reality is that patients with mild disease tend to defer surgery until they are more symptomatic, preferring to continue to epilate.

#### 2 Lash Follicle Destruction Procedures

Procedures to destroy eyelash follicles are used regularly in settings where well-developed ophthalmic services are available and are attractive options in cases where there are only a few misdirected or metaplastic lashes touching the eye in the absence of entropion.^127^ Cryosurgery, electro-epilation, argon laser photocoagulation, and even radiotherapy have been used to ablate lash follicles. Reports of these treatments have generally been small case series with widely varrying outcomes. The variation may be attributed to the number, duration, and power of treatments; disease severity; and the surgeon's ability and equipment. Concerns have also been raised about whether these treatments can provoke or exacerbate the inflammatory process in patients with TT and perhaps even cause the development of trichiasis adjacent to the treatment area.^107,143^ Additionally, the need for expensive equipment and a consistent electricity supply limit their use in trachoma-endemic areas.

##### a Cryotherapy

The aim of cryotherapy (cryosurgery) is to destroy follicles producing aberrant eyelashes by freezing. It was first considered as a treatment option after permanent lash loss was noted as a complication of cryotherapy of eyelid tumors.^16,152^ The procedure requires local anaesthesia and a shield to protect the globe. The cryoprobe (e.g., retinal or Collin's) uses nitrous oxide as the cooling agent. It is placed over the bases of the aberrant lashes. Two cycles of freezing to –20˚C for 20–30 seconds and thawing for 1–2 minutes are carried out through either the conjunctiva or skin. Liquid nitrogen has also been used, but may cause more severe complications unless a very targeted nitrogen spray jet is used.^89^ The lashes are then easily epilated.

Early studies of cryotherapy used a large diameter probe, which caused some damage to surrounding tissues resulting in complication rates as high as 38%.^39,67,118,143^ These included worsening of entropion, lid notching, necrosis, pseudomembrane formation, symblepheron, depigmentation (of particular concern in many trachoma endemic areas), worsening of dry eye symptoms, and activation of herpes zoster ophthalmicus. The size of the probe and the site of application affect the outcome. To improve results, specially designed lid probes were developed that are applied to the tarsal conjunctiva and/or the lid margin rather than to skin. Permanent trichiasis resolution occurs in up to 84% of patients, with minimal complications, including only rare depigmentation.^76,100,109^ The optimal length and strength of treatment and location of treatment (skin, lid margin, or conjunctiva) remains uncertain. Furthermore, the condition specific success rates are unknown as these case series included trichiasis of various etiologies including idiopathic (majority), trauma, trachoma, herpes zoster, congenital distichiasis, and lid margin disease. One trial compared electrolysis (see subsequent section), cryotherapy, and bilamellar tarsal rotation surgery in patients with TT.^106^ The researchers found bilamellar tarsal rotation surgery to have a much better outcome: 80% cure rate compared with 29% and 18% for electrolysis and cryotherapy, respectively. These two lash follicle destruction procedures were limited to one treatment only, however, which is probably insufficient. In addition to less-favorable outcomes, cryotherapy requires specialized equipment that is not available in most settings where TT treatment is performed.

##### b Electrolysis

Electrolysis is widely used to treat trichiasis in settings with well-established ophthalmic services, usually for cases with relatively few metaplastic lashes and no entropion. A fine needle is passed under magnification along the side of the shaft of aberrant lashes into the follicle to a depth of approximately 2.5 mm. A low-power alternating current delivered through a metallic resistor produces heat, which destroys the follicle.^17,51^ The lash is then removed. High recurrence rates have been described from electro-epilation,^106^ but electro-epilation equipment has evolved considerably in recent years and now allows accurate targeting of individual lash follicles with minimal collateral damage.^73^ A non-concurrent prospective study compared electro-epilation and tarsal rotation surgery in 744 cases in Oman (603 patients followed up). A recurrence rate of 51% was found after a single electro-epilation treatment, compared to 62% with surgery (relative risk, 1.22; 95% confidence interval [CI], 1.06–1.41; p value not stated).^82^ Unfortunately the baseline severity was not reported for these patients; but it is probable that electro-epilation was carried out on patients with less severe disease than those who underwent tarsal rotation surgery, making direct comparison impossible.

Radiosurgery is similar to electroysis. Heat is generated by converting alternating current to direct current in the radiofrequency range of the electromagnetic spectrum, using an instrument such as an Ellman Surgitron.^81^

##### c Laser

Argon laser (488–515 nm) is absorbed by melanin, which converts the energy into heat and burns nearby tissue.^77^ Argon laser has been used to destroy lash follicles in patients with trichiasis with success rates ranging from 28–89%.^4,18,72,99,129,149^ A power of 1.0–2.5 watts, for 0.2–0.5 seconds in a spot size of 50–100 μm, is used to create a crater deep enough to burn the follicle. Multiple treatments are required, but only minor complications such as dimpling and mild hypo-pigmentation are described.^99^ In a study of the histopathological changes in rabbit eyelids following treatment with either cryosurgery, electro-epilation or argon laser, the laser produced the most targeted follicle destruction.^12^

##### d Radiotherapy

Similar to cryotherapy, the treatment of trichiasis with radiation resulted from the observation of permanent lash alopecia complicating eyelid tumor irradiation.^37,97^ External beam radiotherapy has only occasionally been used for treating TT.^66^ It has also been tested in rabbits to assess the dosage required to produce lash alopecia.^66^ Topical anaesthetic is required and a thin strip of lead is placed under the eyelid to protect the globe. Multiple irradiations of around 300–600 rad each, to a total of approximately 4,000 rad, are required. This treatment cannot be directed at individual lashes. Multiple complications have been described in reports of irradiation of lid tumors, including conjunctival leukoplakic plaques, scarring and atrophy of the lid, telangiectasis, ectropion, temporary erythema, and dermatitis.^37,54,66,84,91,97^ It is unlikely to be acceptable to most patients,and is not suitable for trachoma endemic settings.

#### 3 Surgical Excision of Aberrant Lash-bearing Follicles or Tissue

Full thickness wedge resections of the affected area are rarely used since the development of less radical tarsotomy procedures. Procedures have been described to surgically remove individual follicles in cases of trichiasis without entropion. The aberrant follicles are accessed either posteriorly through the conjunctiva of an everted lid or anteriorly through the tarsus, which is exposed with a gray line split. A vertical incision is then made through the tarsus along the shaft of the follicle until the lash root bulb is identified, which can then be electrolyzed or cauterized under direct visualization with minimal collateral damage. These procedures require high magnification, are time-consuming and technically challenging, and there are no comparisons of outcomes with other treatments in the literature.^36,142^

### B Corrective Lid Surgery

Surgery to correct TT is a key component of all trachoma blindness control programs in endemic countries. Many techniques have been used. In most the principle is to mobilize the entropic component of the eyelid, then reposition and suture this in a correct orientation to prevent eyelashes scratching the cornea. Tarsal rotation procedures are the most widely used in endemic countries. Some subtle variations in these procedures have developed. Other procedures are rarely used in endemic settings and are usually only performed by ophthalmologists.

#### 1 Tarsal Rotation Procedures

##### a Bilamellar Tarsal Rotation

**Fig. 3:**
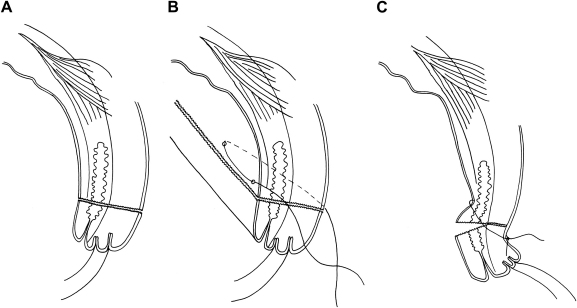
Bilamellar tarsal rotation: *A*: Bilamellar incision. *B*: Horizontal mattress suture. *C*: Postoperative lid eversion.

The bilamellar tarsal rotation (BLTR; Fig. 3) is very similar to the Weiss procedure used for entropion of the lower lid and was first described by Ballen as a treatment for upper lid entropion.^8,139^ In BLTR the lid margin is usually held with hemostats at its medial and lateral ends, or with sutures running through the lid just lateral to the punctum and just medial to the angle. A corneal shield is placed behind the lid. Specially designed lid clamps that indicate where to make the horizontal incision have been developed to provide improved hemostasis and a more standardized placement of the incision.^59,75,77,131^ A full thickness incision is made through the skin, 3 mm above and parallel to the lid margin for a length of about 20 mm (Fig. 3).^8,104,139^ The WHO TT surgery manual states that the incision should be made in two stages: first through the anterior lamella and second, after everting the lid, through the posterior lamella to meet the first.^104^ Others have suggested staggering the two components, with the anterior lamellar incision being 4 mm from the lid margin and the posterior lamellar incision being 2.5 mm from the margin.^111^ A further variation is to make a second horizontal tarsal plate incision in the superior tarsus, to form a bipediculed tarso-conjunctival bridge completely free from Müller muscle.^15^ Three horizontal mattress sutures are inserted that run from the tarsal conjunctiva of the proximal lid fragment, through the anterior lamella component of the distal fragment, to emerge just above the lash line. When these sutures are tightened and tied, the distal fragment of the eyelid is held in an everted position. Silk (4-0) is the most widely used suture material and must be removed at 7–10 days postoperatively. Polyglactin-910 (Vicryl) (5-0) has been shown to be equally effective and can be left to dissolve in situ. ^102a^ The external wound is usually closed with skin sutures.

##### b Posterior Lamellar Tarsal Rotation

**Fig. 4:**
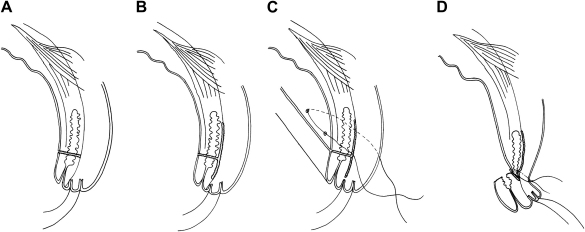
Posterior lamellar tarsal rotation. *A*: Posterior lamellar incision. *B*: Dividing anterior and posterior lamellae. *C*: Horizontal mattress sutures. *D*: Postoperative lid eversion.

There are several operations that combine tarsotomy (incision of conjunctiva and tarsal plate) with everting sutures.^62,63,147^ As a group these are usually referred to as posterior lamellar tarsal rotation (PLTR) procedures (Fig. 4). They vary in their degree of dissection of the anterior and posterior lamellae and in the positioning of the everting sutures. They are performed with the lid in an everted position, usually rotated over a lid plate (Trabut type). A horizontal incision is made through the posterior lamella (conjunctiva and tarsal plate) 3 mm above and parallel to the lid margin. The anterior and posterior lamellae of the proximal (upper) portion are separated by blunt dissection between the orbicularis oculi and the tarsal plate. Similarly, blunt dissection is performed between the lamellae of the distal portion until the dark bulbs of the lash follicles just become visible. Three horizontal everting mattress sutures are passed through the tarsal conjunctiva and plate of the proximal portion of the posterior lamella, then between the divided posterior and anterior parts of the distal fragment, and finally out through the distal anterior lamella, emerging through the skin approximately 3 mm above the lid margin. As the sutures are tightened the proximal segment of the posterior lamella is drawn down and tucks in behind the distal segment of the posterior lamella and the lid everts. The degree of eversion, generally ranging from about 90° to 180°, is determined by how superior in the proximal fragment, and how close to the lid margin of the distal fragment the sutures are positioned.

A greater degree of external rotation of the distal fragment can be achieved by two additional short vertical incisions across the distal portion of the posterior lamella to the lid margin. These are done at both ends and perpendicular to the main incision. In Kettesy's PLTR procedure the sutures emerge through the lid margin and create an approximately 90° rotation.^80^ In the PLTR procedure described by Trabut, and widely used in francophone African countries, the sutures emerge just superior to the lash line, and the distal portion is everted through 180°.^126^

Other variations of the PLTR include continuous sutures, mixture of continuous and mattress sutures, rubber bolsters to tie sutures over, combining PLTR with a tarsal advance (see subsequent section) and/or disinsertion of Müller muscle, and combining posterior tarsal fracture (without dissection between the lamellae) with a shallow gray line split.^42,63,78,93,105,150^

#### 2 Tarsal Advance Procedures

##### a Tarsal Advance

In the tarsal advance procedure the anterior and posterior lamellae are separated by an incision through the gray line and continued superiorly to the full height of the tarsal plate: a lamellar division.^78^ The tarsal plate is then advanced while the anterior lamella is retracted. Horizontal mattress sutures running from the upper fornix to the skin crease, slanting diagonally downwards, raise the terminal edge of the anterior lamella in relation to the anterior tarsal surface. The bare area of anterior tarsal surface produced can be left to granulate or is covered with buccal mucosa.^47,132^ An additional vertical incision through skin and orbicularis about 10 mm from the lateral canthus is meant to facilitate dissection and advance.^90,132^ Additionally, there may be fibrosis and shortening of the levator complex. To release this, the levator complex can be divided and fibrotic Müller muscle excised or recessed to allow increased mobility of the tarsus.^77^

##### b Tarsal Advance and Rotation

**Fig. 5:**
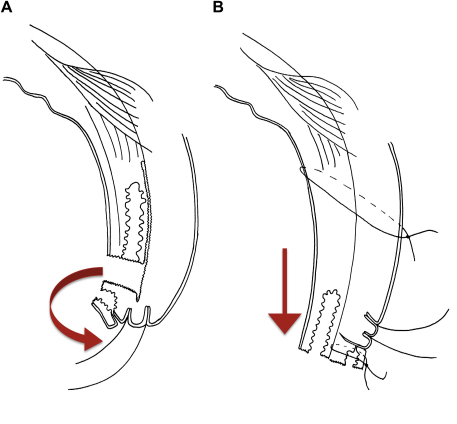
Tarsal advance and rotation. *A*: Posterior lamellar incision and division between posterior and anterior lamellae (arrow indicates 180° rotation of terminal tarsus). *B*: Rotation and suturing of terminal tarsus, inferior advancement and suturing of posterior lamella (arrow indicates inferior movement of posterior lamella).

The tarsal advance and rotation (Collin's modification of the Trabut procedure) is a combination of the posterior lamellar tarsal rotation and the tarsal advance (Fig. 5). Unlike the tarsal advance, however, the anterior and posterior lamellae are separated via the posterior tarsotomy, rather than via the gray line incision.^45,78^ A horizontal mattress suture holds the posterior lamellar portion in an everted position, and a second horizontal suture is placed across the lid running from the upper fornix diagonally downwards to emerge through the skin. When tightened, this second suture raises the anterior lamella.^113^ The tarsal advance and rotation is sometimes combined with dissection of the levator aponeurosis and Müller muscle off the tarsal plate.^78^ This procedure is sometimes used in trachoma endemic settings when there is a degree of lagophthalmos.

#### 3 Posterior Lamellar Lengthening Procedures

Severe cicatricial entropion is characterized by marked shortening of the posterior lamella. In order to achieve lid closure, it is sometimes necessary to lengthen the posterior lamella with grafted tissue. A number of different graft materials have been used: nasal septal cartilage, buccal mucosa, palatal mucosa, donor sclera, contralateral tarsoconjunctiva, and auricular cartilage (overlaid with a mucous membrane).^13,14,33,48,70,110,114,119,122^ Typically the procedure involves a posterior tarsotomy, lamellar division, and suturing of the graft material between distal and proximal tarsal fragments. The technical complexity of these procedures precludes their use in trachoma endemic areas where most TT surgery is performed by non-ophthalmologists.

#### 4 Anterior Lamellar and Lid Margin Procedures

##### a Anterior Lamellar Repositioning

Anterior lamellar repositioning is suitable for mild upper lid entropion without keratinization of the lid margin. A horizontal incision is made through the skin crease to expose the tarsal plate. The anterior lamella is separated from the tarsal plate inferior to the incision line, to the level of the lash roots.^128^ Several sutures are then passed through the skin 1 or 2 mm above the lashes, through the tarsal plate a few mm above the skin bite and then back through the skin, which when tightened externally rotate the lower end of the lid. A shallow vertical gray line incision can be added if a greater degree of eversion is desired. The procedure can also be combined with excision of a strip of orbicularis oculi in the horizontal plane of the skin incision.^71^ Anterior lamellar repositioning can also be combined with aberrant lash follicle excision, which has been reported to give good outcomes.^141^

##### b Eversion Splinting

Eversion splinting involves a shallow gray line incision (to a depth of 3 mm) and external rotation of the distal anterior lamella, which is maintained with three sutures. The sutures run from the lid margin up through the anterior lamella to the tarsal conjunctiva in the upper fornix and back through the skin 6 mm above the lid margin. The sutures are usually tied over a paraffin gauze roll. Some surgeons report low recurrence rates (2–13%) with eversion splinting.^5,140^ In the only randomized controlled trial using this treatment for TT, however, the results were poor (71% TT recurrence).^105^ In variations of this procedure, graft material such as skin, donor sclera, or buccal mucosal membrane are sutured into the gray line split to help maintain the external rotation of the distal anterior lamella.^6,65,116,124,125,130^

##### c Tarsal Grooving (Tarsal Wedge Resection / Cuenod-Nataf/ Anterior Tarsotomy)

This operation, which has several forms known by different names, is a variant of the anterior lamellar repositioning procedure with a horizontal wedge resection of the tarsus and usually no dissection of the anterior lamella from the tarsal plate.^38,43,55,71,94,115,117,121,147^ An incision is made along the skin crease and a variable amount of excess skin removed. The tarsal plate is exposed and incised from the anterior surface, either with two parallel, sloping incisions to form a V-shaped groove (tarsal grooving) or with a single incision to fracture the tarsal plate (anterior tarsotomy). The gray line is usually split and Müller muscle may also be disinserted from the upper end of the tarsal plate. Everting sutures are placed across the groove and out through the skin. As these are tightened, the groove closes, everting the lid margin. The sutures can be tied over a rubber bolster.^32^ The procedure has been combined with dissection of the anterior lamella from the tarsal plate and with division of the orbital septum from the levator aponeurosis.^45,101^ In recent decades this procedure has mainly been used by the trachoma control program in Vietnam. Alternative approaches to tarsal grooving have been described that expose the anterior tarsal surface by extending the gray line split to divide the lamellae.^123^

#### 5 Tarsectomy

Tarsectomy, excision of some or all of the tarsal plate, has been used to treat TT where there is severe scarring and retraction of the tarsus, often associated with lagophthalmos.^11,64,112^ Tarsectomy has been combined with levator recession to help overcome severe lid closure defects.^20,77^ There are concerns about potential complications (e.g., lagophthalmos) of this procedure.

## VIII Outcomes of Trachomatous Trichiasis Treatment

### A Trichiasis Recurrence

Recurrent trichiasis, usually defined as one or more lashes touching the globe in the primary position of gaze, is the most commonly reported outcome measure in TT surgery studies. Recurrence rates from trachoma endemic regions vary widely, ranging from 7.4% at 1 year to 62% at 3 years (Table 3).^1,2,21,29,30,49,82,92,94,96,103,105,106,138,153,154^ Variable follow-up periods can significantly influence the recurrence rate; overall, however, there appears to be a consistent pattern. There is an early peak within the first 3 to 6 months, probably the result of surgery-related factors causing the lid to be incompletely rotated and also to wound healing. This is then followed by an accumulation of later recurrence that may be caused by entropion and new metaplastic changes from progressive cicatricial disease.^30,103^

Multiple risk factors for trichiasis recurrence have been identified, broadly divided into surgeon and surgery related and patient specific. Although some factors, such as pre-operative disease severity, have repeatedly been shown to affect outcome, others are identified on single, usually cross-sectional analyses and require further investigation.

#### 1 Surgery and Surgeon-related Factors

##### a Surgical Procedure

The choice of surgical technique is critical. In trachoma-endemic countries surgery is primarily performed by nurses or eye-care workers who have received short, intensive training in a single surgical procedure. This procedure must be both simple to execute and have low recurrence rates. Three randomized trials have compared procedures in these settings (Table 3, part G). The first study randomly allocated 165 patients with major TT (>5 trichiatic lashes) to one of five different procedures.^105^ It found a recurrence rate of 29% in BLTR, 54% in tarsal advance and rotation, 71% in eversion splinting, 73% in tarsal advance, and 89% in tarsal grooving. The number of procedures in each group was small, and therefore confidence intervals for recurrence rates wide, but BLTR was found to have significantly less recurrence than eversion splinting (p < 0.01), tarsal advance (p < 0.001), and tarsal grooving (p < 0.01), but not less than tarsal advance and rotation. The second study randomly allocated 200 eyelids with major TT without lagophthalmos to either BLTR or tarsal advance and rotation.^106^ BLTR had significantly less recurrence than tarsal advance and rotation (relative hazard, 3.1; 95% CI, 1.9–5.2; p value not stated). The third study followed up 237 eyelids (92.6% of those operated on) that had been randomly allocated to either BLTR or PLTR three months after surgery.^1^ There was no statistically significant difference in recurrence rate between the two procedures (BLTR recurrence, 3/29 [10%]; PLTR recurrence, 2/41 [5%]; p = 0.686) and major trichiasis (BLTR recurrence, 9/86 [10%]; PLTR recurrence, 2/41 [16%]; p = 0.286).

Numerous case series and retrospective reviews have reported on the different procedures. The results of these reports are included in Table 3. They frequently present exceptional results, which are often better than the results of the randomized controlled trials discussed herein. These procedures, however, have usually been carried out in hospitals by ophthalmologists and are often subject to selection bias and follow-up bias.

##### b The Surgeon

The surgeon's technical ability is critical in determining results. Surgeon-specific TT recurrence rates ranged from 0–83% in one study in Gambia.^30^ Other studies have identified significant differences in recurrence rates in different districts, which may also reflect variations in surgeon ability.^82,134^ Although a uniform surgical procedure has been used by all surgeons in each of these studies, subtle differences in incision length and placement and suture tightness may have made substantial differences to the outcome. Incision length has been studied: The recurrence rate at 6 weeks post-BLTR surgery is significantly higher with an incision length <22 mm (crude odds ratio [OR], 3.58; 95% CI, 1.39–9.23).^60^ However, short incisions may be a confounder for small eyelids, which may have higher recurrence rates as the surgery can be more difficult. Left eyelid surgery has been reported to have a higher recurrence rate (32%) than right lid surgery (25%; OR 1.5; 95% CI, 1.0–2.1; p = 0.05), suggesting that left eye surgery is harder for right-handed surgeons and further emphasising the importance of operative technique.^92^ The inter-surgeon variation highlights the need to audit results, supervise surgeons, and retrain where necessary.

##### c Other Factors

Other surgical factors which have been associated with recurrent TT but require further investigation include the type of suture material used,^49^ the use of more than three sutures,^49^ and the need to make postoperative adjustments to the sutures.^123^

#### 2 Patient-specific Factors

##### a Pre-operative Disease Severity and Recurrent Disease

Severe pre-operative disease has consistently been observed to be a risk factor for postoperative TT recurrence.^2,29,30,94,103,133,138,140,153,154^ This may be explained by the shorter, more scarred posterior lamella in severe disease producing residual entropic forces after surgery, and by the increased technical difficulty of operating on such lids.

Similarly, repeat TT surgery is associated with higher recurrence rates. These lids are already self-selected as having had more severe TT surgery at baseline, and repeat surgery is often more challenging as the tissue planes are distorted and scarred.^106,123^

##### b Infection

The role of *C. trachomatis* infection in recurrent trichiasis remains uncertain.^35^ Some studies found an association between *C.trachomatis* infection or living with children with active infections and recurrent trichiasis, whereas others did not.^30,134,154^ Three studies have assessed the effect of postoperative azithromycin treatment on TT recurrence. One of these found a modest effect and two showed no effect.^30,138,153^ The different outcomes may have resulted from the different settings in which the trials were conducted. The study that found a beneficial effect was conducted in a high-prevalence setting (Ethiopia), and the others were not (Gambia and Nepal). The observed beneficial effect on surgical outcome reported in the Ethiopian study does not appear to be explained by differences in *C. trachomatis* infection rates resulting from treatment.^138^ It is possible that azithromycin reduces the load of non-chlamydial bacteria, which may drive chronic tarsal inflammation and scarring, or the anti-inflammatory properties of azithromycin may reduce scarring.

Non-chlamydial conjunctival bacterial infection has been associated with higher rates of recurrence and postoperative inflammation,^29,103^ but it is not known whether this infection drives the scarring process. Infection has been associated with increased expression of potentially important mediators in the scarring process.^26,28^

##### c Other Factors

Several other factors have been associated with TT recurrence: older age^29,30,106,123,134^ (which may be a confounder for more severe disease), female sex,^106,138^ and persistent severe conjunctival inflammation.^30,134^

### B Visual Acuity and Corneal Opacity

Trachomatous visual impairment and blindness, which result from corneal opacification, have generally been thought of as irreversible. Corneal transplantation is rarely available in trachoma-endemic countries, and the results have been disappointing. One case series reported graft rejection in 4 of 7 penetrating keratoplasties performed for trachomatous corneal opacity.^151^ Another reported successful results from ipsilateral kerato-rotation.^19^ As cataract extraction was performed simultaneously, the improvement in visual acuity is difficult to interpret. Additionally, this sort of highly specialized procedure is not suitable for trachoma-endemic regions. Despite this discouraging background, several recent studies have shown modest improvement in visual acuity following trichiasis surgery—about one LogMAR line of acuity.^30,106,144^ This may be the result of a reduction in epiphora and photophobia, an improvement in the quality of the tear film, the resolution of corneal epithelial damage, or a gradual fading of milder corneal scars. Conversely in some patients, the corneal opacity develops or progresses despite successful trichiasis surgery.^30^ The reasons for this are not well understood, but may involve conjunctival inflammation, keratinisation, dry eye, or secondary bacterial infection.

### C Surgical Complications

Serious complications are fortunately relatively rare in TT surgery, even when it is performed in resource-limited settings.

#### 1 Stitch Granulomas

**Fig. 6:**
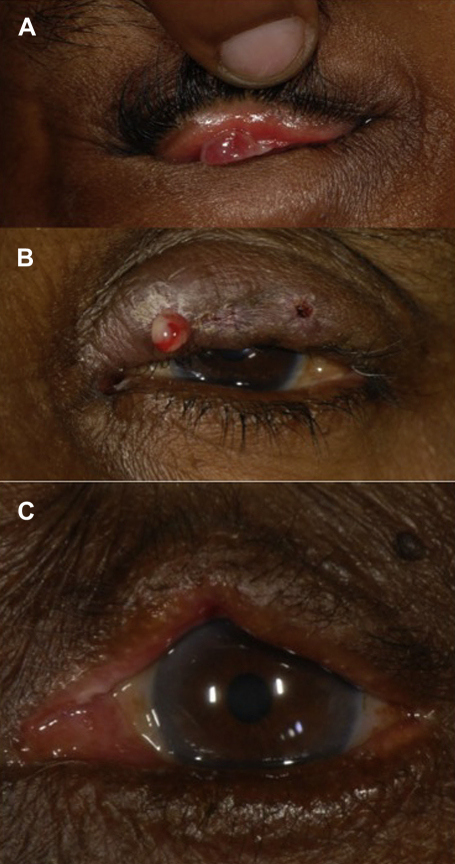
*A*: Post-operative granuloma. *B*: Post-operative wound infection *C*: Postoperative lid notching.

Stitch granulomas (Fig. 6A) have been reported to occur with most TT surgical techniques and with the use of both non-absorbable and absorbable sutures.^1,2,6,23,45,60,96,106,125^ The highest reported rates are 14%. These can be quite large—even obscuring the visual axis.^2^ The treatment of granulomas vary. Some surgeons leave them in situ to regress spontaneously, others excise them and others use steroid drops.^79,125^ In general, large granulomas should be removed and smaller ones may be left if not causing discomfort.

#### 2 Wound Infection

Despite the frequent infection of the conjunctival sac associated with trichiasis, wound infections are surprisingly infrequent after TT surgery (Fig. 6B).^1,30^ Preparing the skin and the conjunctival sac with a suitable antiseptic solution such as povidone iodine is very important. Similarly, postoperative topical antibiotics may also reduce the risk.

#### 3 Lid Notching and Overcorrection

Lid notching occurs when one of the everting sutures is over-tight, producing an irregular lid contour (Fig. 6C). Rates as high as 6.3% have been reported.^21^ If notching is visible at the end of surgery, the relevant suture should be adjusted or replaced. Overcorrection of the whole lid is less common and may be less noticeable if bilateral. In the absence of lagophthalmos, mild to moderate overcorrection can be left, as it tends to settle back into a more anatomically correct position during the following months. More severe overcorrection may require surgical revision.

#### 4 Other Complications

Intra- and postoperative hemorrhage occurs occasionally, usually when the marginal artery has been cut. Diathermy is rarely available in trachoma endemic settings, but bleeding can usually be controlled by prolonged pressure. Serious, uncontrollable bleeding is extremely rare in the absence of other hematological disorder.

## IX Treatment of Trachomatous Trichiasis in Endemic Settings

### A Choice of Operation

In the light of the results from the trials described herein and the relative simplicity of the BLTR procedure, WHO advocates the use of this operation for all patients with TT without lagophthalmos in endemic countries, irrespective of the amount of trichiasis and severity of the entropion.^104,148^ Various forms of PLTR are widely used in trachoma-endemic regions, and this is generally considered an acceptable alternative. The only direct comparison between these two techniques showed similar outcomes, although the surgeries were performed by ophthalmologists in a teaching hospital, and there was only three months of follow-up.^1,147^

Lid shortening and lagophthalmos are often found in individuals with severe conjunctival scarring or following previous surgery.^77,78^ Both the BLTR and PLTR procedures can cause lid shortening and are therefore not suitable for eyes with lagophthalmos. In cases of significant lagophthalmos the shortened posterior lamella can be mobilized, allowing it to advance, by recession or release of the levator and Müller muscles.^77,78^ This can be combined with a Trabut-type operation and is referred to as the Tarsal Advance and Rotation in the WHO surgery manual, which recommends its use in this situation.^104^ Alternatively, the posterior lamella can be lengthened using graft material, although this is rarely available in trachoma-endemic settings. It remains uncertain which of the various options for the treatment of trachoma-related defective lid closure offers the best results. One trial attempted to compare the Tarsal Advance and Rotation (with or without levator incision) with a buccal mucosal membrane graft into the posterior lamella; unfortunately, there was insufficient enrollment.^106^

### B Indications for Surgery

Currently WHO recommends lid rotation surgery (BLTR or PLTR) for all patients with TT, irrespective of severity. There is a broad consensus that surgery is appropriate for patients with major TT, but for minor TT and particularly those patients with just a few peripheral, metaplastic lashes without entropion, practice varies. Many patients and clinicians prefer to defer surgery and epilate until more problematic disease develops. The rationale for the WHO recommendation is that TT patients may not be seen again; therefore, surgery should be performed when the opportunity arises.

### C Delivering TT Surgery Services in Endemic Countries

#### 1 Who Should Operate?

As a result of the huge burden of un-operated TT and the scarcity of ophthalmologists in trachoma-endemic countries, the vast majority of surgery is done by nurses, health-care workers, or integrated eye care workers (IECWs) with varying degrees of training and experience. The training course is usually 2–3 weeks long. One study has shown similar recurrence rates outcomes comparing ophthalmologists and IECWs.^2^ That study, however, only compared two IECWs with two ophthalmologists, all of whom had very high output. Another study has reported the not-unexpected finding that there is marked variation in the rates of recurrence between different surgeons.^30^ Suitably trained health-workers and/or ophthalmologists can provide TT surgery, but they should be performing TT surgery regularly and have their outcomes audited from time to time. Unfortunately, many TT surgeons perform only a few procedures per year.^61,86^ Surgeons frequently reported having a shortage of time, as many also manage vaccination programs, childbirth, and diseases such as tuberculosis, acquired immune deficiency syndrome, and malaria. Additionally, many work in rural clinics where health-system failures prevent them from receiving the necessary equipment and consumables.^61^ Furthermore, many trained TT surgeons stop doing TT surgery altogether; a study from Ethiopia found a surgeon attrition rate of 59% in the most trachoma-prevalent region of the country.^61^

#### 2 How Should the Service Be Structured?

There are two broad models for TT surgical provision: health-center-based (“static” or “fixed” services) and outreach (“campaign”). In the health-center model, TT surgery is integrated into routine clinical services. The catchment population is educated about the service and patients are expected to attend the clinic for assessment and treatment as required. In the outreach model a temporary clinic and operating theater are set up for a short period of time, usually in a rural setting without pre-existing facilities. The “campaign” is advertised to the local community by health-care workers, in markets and religious centers, by radio and word of mouth. There are advantages and disadvantages to each approach. A successful static model may be more sustainable in the long term, but depends on a well-organized health system that can ensure that the correct equipment, personnel, and patients are in the clinic at the same time. The outreach model enables large numbers of operations to be conducted in a short period of time, often in locations where patients have not been able to access TT surgery, but usually depends on charitable organizations to fund and organize the service and does not build a sustainable health system. Studies from Ethiopia and Tanzania have found that despite training of large numbers of TT surgeons and provision of equipment for a clinic-based service, the bulk of the surgery has been performed in outreach campaigns.^61,86^ Additionally, surgical uptake is usually higher in village surgical campaigns than in health-center-based surgery (66% vs. 44%; rate ratio, 1.49; 95% CI, 1.11–2.01; p = 0.009), with no significant difference in outcomes.^24^ In high-prevalence TT settings, both approaches are likely to be required.

#### 3 Barriers to Surgery

Studies examining barriers to surgery are presented in Table 4. The acceptance of surgery is repeatedly shown to be poor, with 18% and 66% of patients having surgery, even when free transport and surgery are provided.^22,24,40,87,98,102,136^ Despite the different settings of these studies, common reasons emerge for failure: Logistical barriers include distance to surgery, lack of transport, lack of escorts (the majority of TT patients have bilateral disease and bilateral surgery, making an escort essential), indirect costs (food, accommodation, transport, and paying someone to cover work and/or home duties), and lack of time. Additionally, when the symptoms are not severe, self-administered epilation seems a more desirable treatment, particularly in the context of the poor outcomes reported from many field trials. There may be health-system problems, including failure to inform patients about existing TT surgical services and equipment/personnel failures that prevent surgery on eligible patients attending clinic.^61^ Many of these barriers can be overcome by moving the surgery closer to the patient, but this may be expensive and require complicated planning. The highest uptake rate (66%) is achieved by providing surgery at a village level.^24^

#### 4 The Cost of Treatment

During a 30-year program of surgical treatment in Burma, the average cost per case of visual impairment prevented by surgical treatment has been calculated as US$ 193, although this has become much cheaper in the last ten years (US$ 41), presumably because of increased efficiency and decreased capital costs.^52^ The cost effectiveness of trachoma surgery in seven regions of the world was calculated as between US$ 13 and US$ 78 per disability-adjusted life year (DALY) averted and was cheapest (US$ 13–17) in Africa, where the greatest burden lies.^9^ In Gambia, each operation costs US $6.13, whereas the estimated life-time loss of productivity is estimated to be US$ 89.^57^ These figures compare favorably to other ophthalmic operations. For example the cost effectiveness of cataract surgery in resource poor settings has been estimated to range great from US$ 9/quality-adjusted life year (QALY) to US$ 1,600/QALY.^85^

## X Conclusion

Trachomatous trichiasis causes visual loss and blindness and remains a major public health problem in many low-income countries. Surgery is the mainstay of treatment and is often effective. Recurrence rates can be high and uptake low, and there remains uncertainty about whether all patients with minor TT require surgical treatment. A multitude of surgical procedures has been devised and tried. Bilamellar tarsal rotation and posterior lamellar tarsal rotation are currently the procedures of choice, as they are relatively quick and easy to teach and perform and have lower recurrence rates. Further research needs to be conducted into optimal surgical techniques and training and alternatives for treating minor trichiasis.

## XI Method of Literature Search

Articles pertaining to trachomatous trichiasis and trichiasis surgery were sought using Medline (all years). The following search terms were used: *trachomatous trichiasis, trachoma and trichiasis, trachoma and surgery and barriers, trachoma and opacity, trichiasis and history, trachoma and history*. Non-English literature was translated into English.

## Disclosure

The salaries of two of the authors of this paper (S.R. and M.B.) were funded by grants from the Band Aid Foundation with Fight For Sight and The Wellcome Trust (080741/Z/06/Z). The funding organizations had no role in the design or writing of this article. The authors reported no proprietary or commercial interest in any product mentioned or concept discussed in this article.

## Figures and Tables

**Table 1:** The WHO Trachoma Grading System (FPC)

Grade	Description
Upper Tarsal Follicles (F)	
F 0	No follicles.
F 1	Follicles present, but no more than 5 in zones 2 and 3 together.
F 2	More than 5 follicles in zones 2 and 3 together, but less than 5 in zone 3.
F 3	Five or more follicles in each of the three zones.
Upper tarsal papillary hypertrophy and diffuse inflammation (P)	
P 0	Absent: normal appearance
P 1	Minimal: individual vascular tufts (papillae) prominent, but deep subconjunctival vessels on the tarsus not obscured.
P 2	Moderate: more prominent papillae, and normal vessels appear hazy, even when seen by the naked eye.
P 3	Pronounced: conjunctiva thickened and opaque, normal vessels on the tarsus are hidden over more than half of the surface.
Conjunctival scaring (C)	
C 0	No scarring on the conjunctiva.
C 1	Mild: fine scattered scars on the upper tarsal conjunctiva, or scars on other parts of the conjunctiva.
C 2	Moderate: more severe scarring but without shortening or distortion of the upper tarsus.
C 3	Severe: scarring with distortion of the upper tarsus.
Trichiasis and/or entropion (T/E)	
T/E 0	No trichiasis and/or entropion.
T/E 1	Lashes deviated towards the eye, but not touching the globe.
T/E 2	Lashes touching the globe but not rubbing the cornea.
T/E 3	Lashes constantly rubbing the cornea.
Corneal scarring (CC)	
CC 0	Absent.
CC 1	Minimal scarring or opacity but not involving the visual axis, and with clear central cornea.
CC 2	Moderate scarring or opacity involving the visual axis, with the papillary margin visible through the opacity.
CC 3	Severe central scarring or opacity with the papillary margin not visible through the opacity.

**Table 2:** The WHO Simplified System for the Assessment of Trachoma

Grade		Description
Trachomatous inflammation – follicular	TF	The presence of five or more follicles (>0.5 mm) in the upper tarsal conjunctiva
Trachomatous inflammation – intense	TI	Pronounced inflammatory thickening of the tarsal conjunctiva that obscures more than half of the deep normal vessels
Trachomatous scarring	TS	The presence of scarring in the tarsal conjunctiva
Trachomatous trichiasis	TT	At least one lash rubs on the eyeball
Corneal opacity	CO	Easily visible corneal opacity over the pupil

**Table 3:** Studies Examining Recurrence Rates after TT surgery

Author	Study Description	Results	Comments
**Part A: BLTR and variants**			
Khandekar et al, 2001^82^	**Design:** non-concurrent prospective cohort study **Procedure:** BLTR **Location:** Oman **Number of patients:** 292 **Number of eyelids:** Not stated in paper **Case severity:** Major TT **Surgeon grade:** not stated **Follow-up period:** mean: 3.1 yrs (range 2–3.5 yrs) **Outcome measure:** recurrent TT (1+ lashes)	**Follow-up rates:** 81% (603/744) **Recurrence rates:** 61.8% overall 43.6% Major TT recurrence 18.2% Minor TT recurrence **Associations with recurrence:** • District • Duration of follow-up • Infective conjunctivitis at follow-up **Complications:** none stated	Electro-epilation for Minor TT also studied: recurrence rate 50.6%

Alemayehu et al, 2004^2^	**Design:** RCT of surgery done by (A) ophthalmologists vs. (B) IECW **Procedure:** BLTR **Location:** Ethiopia **Number of patients:** 982 **Number of eyelids:** 1,750 **Case severity:** Minor and Major TT **Surgeon grade:** ophthalmologists and IECW **Follow-up period:** 3 months and 6 months **Outcome measure:** recurrent TT (1+ lashes)	**Follow-up rates:** 77% at 6 months **Recurrence rates:** • By person: 14.3% (124/865) by 6 months • By eye: 9.0% (140/1553) by 6 months • Arm A: 12.7% (47/370) of people at 3 months • Arm B: 9.9% (34/343) of people at 3 months **Associations with recurrence:** baseline severity **Complications:** • Granulomas: 14% (100/713) • Lid contour abnormality: 6.2% (44/713)	No significant difference in TT recurrence rates found between ophthalmologists and IECW. Leading study supporting the use of non-ophthalmologists in the provision of TT surgery services.

Zhang et al, 2004^154^	**Design:** Prospective case-control cohort study **Procedure:** BLTR **Location:** Nepal **Number of patients:** 78 **Number of eyelids:** 79 **Case severity:** Minor and Major TT **Surgeon grade:** not recorded **Follow-up period:** 12 months **Outcome measure:** recurrent TT (1+ lash)	**Follow-up rates:** 56% at 12 months **Recurrence rates:** 25% (11/44) **Associations with recurrence:** • Presence of *C. trachomatis* • >5 trichiatic lashes at baseline • TI or TF **Complications:** none	

Merbs et al, 2005^92^; West et al, 2005^134,a^	**Design**: Retrospective cohort study **Procedure:** BLTR **Location:** Tanzania **Number of patients:** 384 **Number of eyelids:** 630 **Case severity:** not recorded **Surgeon grade:** not recorded **Follow-up period:** variable. All >18/12 **Outcome measure:** recurrent TT (1+ lash or evidence of epilation)	**Follow-up rates:** 64% (384/601) **Recurrence rates:** 27.9% (176/630) **Associations with recurrence:** • District • TI in the surgical eye • 2 or more household members with TI • Older age • >1 child in house with *C.trachomatis* infection • Left eye **Complications:** none recorded	

El Toukhy et al, 2006^49^	**Design:** Prospective observational **Procedure:** BLTR **Location:** Egypt **Number of patients:** 493 **Number of eyelids:** 638 **Case severity:** Minor and Major TT **Surgeon grade:** ophthalmologist **Follow-up period:** 8–10 weeks **Outcome measure:** surgical failure (1+ lash)	**Follow-up rates:** 94% (599/638) **Recurrence rates:** 16.4% (98/599) **Associations with recurrence:** • Pre-operative corneal opacity • Pre-operative corneal staining • Use of silk sutures (rather than Vicryl) • Use of four or more sutures **Complications:** none recorded	

Zhang et al, 2006^153^	**Design**: RCT of post-op treatment (A) azithromycin vs. (B) placebo **Procedure:** BLTR **Location:** Nepal **Number of patients:** 109 **Number of eyelids:** 148 **Case severity:** Minor and Major TT **Surgeon grade:** ophthalmologist **Follow-up period:**12 months **Outcome measure:** recurrent TT (1+ lash or evidence of epilation)	**Follow-up rates:** 78% (116/148) **Recurrence rates:** 29% (33/114) at 12 months **Associations with recurrence:** • Major TT at baseline associated with recurrence at 3 months • Placebo post-operatively in patients with Major TT at baseline **Complications:** none recorded	There was no significant difference in the overall recurrence rate by treatment arm. For eyes which had Major TT pre-operatively, there was significantly less recurrence in the azithromycin arm at 12 months (21% azithromycin, 62% placebo, p=0.030)

West et al, 2006^138^; Gower et al, 2010^60,a^	**Design:** RCT of postoperative (A) oral azithromycin (B) oral azithromycin for patient and family members (C) topical TTC **Procedure:** BLTR **Location:** Ethiopia **Number of patients:** 1,452 **Number of eyelids:** 1,452 (bilateral TT: 1 eye randomly selected) **Case severity:** Minor and Major TT **Surgeon grade:** IECW **Follow-up period:** 12 months **Outcome measure:** recurrent TT (1+ lash or evidence of epilation)	**Follow-up rates:** 97% (1414/1452) patients **Recurrence rates:** 7.6% (107/1414) overall (A) 5.5% (26/472), (B) 7.6% (36/472), (C) 9.6% 45/470) **Associations with recurrence:** • Male sex • Moderate/severe pre-operative entropion • No azithromycin treatment • Incision < 22 mm **Complications:** • Granuloma: 10.5% • Lid contour abnormalities: 1.2%	The incidence of recurrence was lower in the azithromycin group (A + B) compared to the topical tetracycline group (C): 6.9/100 person-years vs 10.3/100 person-years (P = 0.047), respectively. However *C. trachomatis* not a risk factor for recurrence.

**Part B: PLTR and variants**			
Halasa and Jarudi, 1974^63^	**Design:** retrospective case series **Procedure:** PLTR with rubber bolster **Location:** USA **Number of patients:** 154 **Number of eyelids:** 300 **Case severity:** not stated **Surgeon grade:** not stated **Follow-up period:** minimum: 6 months, maximum: 9 years **Outcome measure:** “recurrence”	**Follow-up rates:** retrospective **Recurrence rates:** 3.3% “severe enough to necessitate re-operation”(10/300). Overall TT recurrence rate not stated. **Associations with recurrence:** none recorded **Complications:** none	

Bog et al, 1993^21^	**Design:** prospective cohort study **Procedure:** PLTR **Location:** Tanzania **Number of patients:** 94 **Number of eyelids:** 156 **Case severity:** Major TT **Surgeon grade:** ophthalmic nurse **Follow-up period:** 9–36 months (mean 25.5 months) **Outcome measure:** recurrent TT (1+ lash)	**Follow-up rates:** 91% (86/94) patients **Recurrence rates:** 17.4% (25/144) lids **Associations with recurrence:** none recorded **Complications:** • 6.3% (9/144) notching • 0.7% (1/144) infection	

Yeung et al, 1997^150^	**Design:** not stated **Procedure:** posterior tarsal fracture + shallow gray line split **Location:** Hong Kong **Number of patients:** 19 **Number of eyelids:** 24 (20 trachomatous aetiology) **Case severity:** not stated **Surgeon grade:** not stated **Follow-up period:** 2–12 months (mean 6.3) **Outcome measure:** recurrent trichiasis (1+ lashes)	**Follow-up rates:** 100% (24/24) **Recurrence rates:** 35% (9/24) **Associations with recurrence:** none recorded **Complications:** 4.2% (1/24): lid closure defect	

Bowman et al, 2000^24a^	**Design:** retrospective cross sectional **Procedure:** PLTR **Location:** Gambia **Number of patients:** 65 **Number of eyelids:** 115 **Case severity:** Major TT **Surgeon grade:** “Medical staff” and “Senior ophthalmic assistants” **Follow-up period:** median:7 (range not stated) **Outcome measure:** recurrent TT (1+ lash)	**Follow-up rates:** retrospective study **Recurrence rates:** • 55% (63/115) eyes • 65% (42/65) people **Associations with recurrence:** none recorded **Complications:** 3.5% (4/115) lagophthalmos	Long term recurrence rates can be very high.

Bowman et al, 2002^23^	**Design:** prospective cohort study **Procedure:** PLTR **Location:** Gambia **Number of patients:** 34 **Number of eyelids:** 54 **Case severity:** Major TT **Surgeon grade:** ophthalmic nurse **Follow-up period:** 12 months **Outcome measure:** number of trichiatic lashes at 12 months F/U	**Follow-up rates:** 98% (53/54) **Recurrence rates:** 28% (15/53). Comprising: • 9% (5/53) Major TT recurrence • 19% (9/53) Minor TT recurrence **Associations with recurrence:** none recorded **Complications:** • 9.4% (8/53) granuloma • 3.8% (2/53) lid notching • 1.9% (1/53) ptosis	Although recurrence rates can be high, this study identified, that 64% (9/14) of the recurrences had <6 lashes.

Burton et al, 2005^29^	**Design:** prospective cohort study **Procedure:** PLTR **Location:** Gambia **Number of patients:** 162 **Number of eyelids:** 214 **Case severity:** not stated **Surgeon grade:** ophthalmic nurse **Follow-up period:** 36-48 months **Outcome measure:** recurrent TT (1+lash)	**Follow-up rates:** 87% (141/162) **Recurrence rates:** 41.6% (89/214) lids (52/214) Comprising: • 24.3% (52/214) Major TT recurrence • 17.3% (37/214) Minor TT recurrence **Associations with recurrence:** • Conjunctival bacterial infection • Papillary inflammation (P2/P3) **Complications:** none recorded	

Burton et al, 2005 (1)^30^; Rajak et al, 2010 (2)^103^	**Design:** (1) RCT of postoperative (A) azithromycin vs. (B) TTC (2) Prospective cohort study **Procedure:** PLTR **Location:** Gambia **Number of patients:** 451 **Number of eyelids:** **Case severity:** Major TT **Surgeon grade:** ophthalmic nurse **Follow-up period:** RCT: 12 months Prospective cohort study: 4 years **Outcome measure:** recurrent TT (1+ lash)	**Follow-up rates:** (1) 94.4% (426/451) (2) 74.7% (266/365) **Recurrence rates** (1) 12 months**:** 41.3% (176/451) • Arm A: 41.2% (84/204) • Arm B: 41.4% (92/222) (2) 4 years (arms A&B combined): 41% (110/266) **Associations with recurrence:** (1) 12 months • >10 trichiatic lashes pre-operatively • Severe conjunctival inflammation (P3) at f/u • Conjunctival bacterial infection at f/u • Surgeon (2) 4 years • >10 trichiatic lashes pre-operatively • Moderate/severe conjunctival inflammation (P2/33) at 4 years **Complications (12 months):** • 0.44% (2/451) defective lid closure • 0.22% (1/451) lid infection	No significant difference in post-operative recurrence for azithromycin and TTC Wide variation in recurrence rates of different surgeons. The recurrence rate does not increase from one year to four years post-operatively

**Part C: Posterior lamella lengthening**			
Hosni, 1974^70^	**Design:** not stated **Procedure:** posterior lamella lengthening with buccal graft **Location:** Egypt **Number of patients:** 708 **Number of eyelids:** 426 **Case severity:** “trachomatous entropion trichiasis” **Surgeon grade:** not stated **Follow-up period:** not stated **Outcome measure:** recurrent TT (1+ lashes)	**Follow-up rates:** 100% **Recurrence rates:** 10.0% (71/708) overall, comprising: 4.0% (28/708) Minor and 6.1% (43/708) Major recurrence **Associations with recurrence:** not recorded **Complications:** none	
**Part D: Tarsal grooving and variants**			
Prachakvej et al, 1978^101^	**Design:** retrospective case series **Procedure:** tarsal grooving + division of orbital septum from levator aponeurosis **Location:** Thailand **Number of patients:** 24 **Number of eyelids:** 35 **Case severity:** “advanced trachomatous entropion” **Surgeon grade:** not stated **Follow-up period:** not stated **Outcome measure:** not stated	**Follow-up rates:** 100% **Recurrence rates:** “undercorrection”: 5.7% (2/35) **Associations with recurrence:** none recorded **Complications:** overcorrection: 2.9% (1/35)	

Thanh et al, 2004^123^; Khandekar et al, 2009^82a,a^	**Design:** prospective cohort study **Procedure:** Cuenod-Nataf **Location:** Vietnam **Number of patients:** 472 **Number of eyelids:** 648 **Case severity:** any TT **Surgeon grade:** ophthalmologist **Follow-up period:** 24 months **Outcome measure:** 1+ trichiatic lashes	**Follow-up rates:** 96% (453/472) **Recurrence rates:** 15.9% (101/636) **Associations with recurrence:** • Age >70 • Female • Surgeon • District • History of previous TT surgery • Severe conjunctival scarring • Post-op adjustments made to suture tension **Complications:** none recorded	
**Part E: Eversion splinting**			
Win, 1976^140^	**Design:** not stated **Procedure:** gray line split **Location:** Burma **Number of patients:** not stated **Number of eyelids:** 1861 **Case severity (author's grading system):** • 128/528 grade I (TT of half length of lid margin, lid soft) • 272/528 grade II (TT of whole length of lid margin, lid soft) • 128/528 grade III (TT of whole length of lid margin, lid hard) **Surgeon grade:** not stated **Follow-up period:** 12 months **Outcome measure:** not stated	**Follow-up rates:** 28% (528/1561) **Recurrence rates:** 8/528 (2%) **Associations with recurrence:** severe pre-op TT **Complications:** none recorded	Very low f/u rate: difficult to draw conclusions on gray line split technique

Thommy, 1980^124^	**Design:** prospective cohort **Procedure:** gray line split with auto-skin graft in incision **Location:** Nigeria **Number of patients:** 200 **Number of eyelids:** 341 **Case severity:** not stated **Surgeon grade:** not stated **Follow-up period:** minimum: 4 months, maximum: 2 years **Outcome measure:** “entropion or misdirected lashes”	**Follow-up rates:** 93.8% (320/341) **Recurrence rates:** 6.6% (21/320) **Associations with recurrence:** none recorded **Complications:** none recorded	

Thommy, 1981^125^	**Design:** not stated **Procedure:** gray line split with scleral graft in incision **Location:** Nigeria **Number of patients:** 136 **Number of eyelids:** 155 **Case severity:** not stated **Surgeon grade:** not stated **Follow-up period:** minimum: 2 months, maximum: 15 months **Outcome measure:** “trichiasis”	**Follow-up rates:** not stated, implies 100% **Recurrence rates:** 7.7% (12/155) **Associations with recurrence:** **Complications:** • Granuloma: 10.3% (16/155) • Partial sloughing of scleral strip: 2.6% (4/155)	

**Part F: Tarsectomy**			
Jones et al, 1976^77^	**Design:** prospective cohort **Procedure:** tarsectomy **Location:** Iran **Number of patients:** not stated **Number of eyelids:** 36 **Case severity:** severe entropion **Surgeon grade:** not stated **Follow-up period:** 12 months **Outcome measure:** entropion, “few aberrant lashes”	**Follow-up rates:** 83.3% (33/36) **Recurrence rates:** 15.2% (5/33) **Associations with recurrence:** none recorded **Complications:** high lid arch: 18.2% (6.33)	

**Part G: >1 procedure**			
Kemp and Collin, 1986^78^	**Design:** retrospective case series **Procedure:** 1. Anterior lamella repositioning +/− gray line split: for minimal entropion 2. Anterior lamella repositioning + gray line split + tarsal wedge resection or lamellar division: for moderate entropion 3. Tarsal advance and rotation or posterior lamellar lengthening procedure: for severe entropion	**Follow-up rates:** retrospective study **Recurrence rates:** 1. Minimal entropion: 31.9% (30/94) 2. Moderate entropion: 26.5% (13/49) 3. Severe entropion: 25% (10/40) **Associations with recurrence:** none recorded **Complications:** none recorded	
	**Location:** UK **Number of patients:** 107 **Number of eyelids:** 183 **Case severity:** not stated. 40.6% TT (73/180) **Surgeon grade:** oculoplastic surgeon **Follow-up period:** Mean 3 years, minimum 10 months **Outcome measure:** need for further surgery		

Babalola, 1988^6^	**Design:** probable retrospective case series **Procedure:** 1. BLTR 2. Gray line split with scleral graft in incision **Location:** Nigeria **Number of patients:** not stated **Number of eyelids:** 1. 31 2. 23 **Case severity:** not stated **Surgeon grade:** not stated **Follow-up period:** not stated **Outcome measure:** not stated	**Follow-up rates:** 100% **Recurrence rates:** 1. 22.6% (7/31) 2. 26.1% (6/23) **Associations with recurrence:** none recorded **Complications:** 1. BLTR Granuloma: 9.7% (3/31) Infection: 3.2% (1/31) 2. Gray line split/scleral graft Graft sloughing/rejection: 30.4% (7/23) Infection: 4.3% (1/23) Secondary hemorrhage: 4.3% (1/23)	

Nasr, 1989^94^	**Design:** retrospective case series **Procedure:** 1. Anterior tarsotomy: for mild to moderate entropion 2. PLTR+/− auricular or scleral graft: for moderate to severe entropion 3. Anterior lamellar recession + mucus membrane graft for severe entropion with irregular eyelid margin **Location:** Saudi Arabia **Number of patients:** 960 **Number of eyelids:** not stated **Case severity:** All entropion severity **Surgeon grade:** ophthalmologist **Follow-up period:** average 22.5 months (range not stated) **Outcome measure:** not stated	**Follow-up rates:** 1. Not stated 2. 92.8% (464/500) PLTR cases 3. Not stated **Recurrence rates (“success [not defined] rates”):** 1. Denominator not stated 2. 11.6% (54/464) 3. c.50% (exact figure not stated) **Associations with recurrence:** severe pre-op entropion **Complications:** none recorded	

Reacher et al, 1990^105^	**Design:** RCT of 5 surgical techniques (see below) **Procedure:** (A) BLTR (B) Tarsal advance and rotation (C) Eversion splinting (D) Tarsal advance (E) Tarsal grooving **Location:** Oman **Number of patients:** 165 **Number of eyelids:** 165 (bilateral TT: 1 eye selected for trial) **Case severity:** Major TT **Surgeon grade:** not stated **Follow-up period:** average 7.9 months (range: 5–11 months) **Outcome measure:** recurrent TT (1+ lashes)	**Follow-up rates:** 92.7% (153/165) **Recurrence rates:** (A) 25.6% (10/39) (B) 50.0% (11/22) (C) 66.7% (14/21) (D) 44.7% (17/38) (E) 87.9% (29/33) **Associations with recurrence:** none recorded **Complications:** defective lid closure	The first RCT of surgical techniques in the field.

Reacher et al, 1992^106^	**Design:** RCT of 3 procedures for Minor TT and 2 surgical techniques for Major TT **Procedure:** 1. Minor TT (A) Electrolysis (B) Cryoablation (C) BLTR 2. Major TT (A) BLTR (B) TA&R **Location:** Oman **Number of patients:** 357 **Number of eyelids:** 384 (bilateral TT: 1 eye selected for trial) **Case severity:** Minor and Major TT **Surgeon grade:** not stated **Follow-up period:** 9-21 months **Outcome measure:** recurrent TT (1+ lashes)	**Follow-up rates:** 94% of lids seen at either/both 9- and 21-month follow-ups **Recurrence rates:** 1. Minor TT (A) 52.6% (30/57) (B) 71.9% (41/57) (C) 11.5% (6/52) 2. Major TT (A) 18.4% (18/98) (B) 45.5% (46/101) **Associations with recurrence:** none recorded **Complications:** Granuloma 12.6% (19/151) of BLTR Poor cosmetic result (ectropion) 5% (5/101) of TA&R 2% (2/98) of BLTR Immediate post-op haemorrhage 1.6% 2/129 TA&R Overcorrection requiring revision 1.3% (2/151) BLTR Defective lid closure: 1% (1/101) of TA&R	Led to The WHO endorsing BLTR for all TT surgery

Negrel et al, 2000^96^	**Design:** retrospective case series of random sample **Procedure:** 1. BLTR (91%) 2. PLTR (9%) **Location:** Morocco **Number of patients:** 740 **Number of eyelids:** 740 **Case severity:** All TT **Surgeon grade:** Ophthalmologists, general doctors, and nurses **Follow-up period:** All >6 months **Outcome measure:** recurrent TT (1+ lashes)	**Follow-up rates:** retrospective study **Recurrence rates:** 15.8% (117/740) **Associations with recurrence:** none recorded **Complications:** • Overcorrection: 2.3% (17/740) • Ptosis: 0.4% (3/740) • Lid necrosis without corneal exposure: 3.6% (27/740) • Lid necrosis with corneal exposure: 0.14% (1/740) • Granuloma: 0.95% (7/740)	

Adamu et al, 2002^1^	**Design:** RCT of surgical techniques **Procedure:** (A) BLTR vs. (B) PLTR **Location:** Ethiopia **Number of patients:** 153 **Number of eyelids:** 256 (BLTR: 124, PLTR: 132) **Case severity:** All TT **Surgeon grade:** ophthalmologist **Follow-up period:** 3 months **Outcome measure:** recurrent TT (1+ lashes)	**Follow-up rates:** 92% (141/153) patients **Recurrence rates:** (A) 10.4% (B) 12.3% **Associations with recurrence:** none recorded **Complications:** • 3.48% (4/115) over-correction in BLTR • 1.61% (2/124) post-op bleeding in BLTR • 0.81% (1/124) post-op infection in BLTR • Notching and granuloma occurred more frequently in BLTR than PLTR (data not given)	The only randomized comparison of the two most widely used procedures. It found no significant difference in recurrence rates for Minor (p=0.686, OR and C.I. not stated) or Major (p=0.286 OR and C.I. not stated) TT.

Dhaliwal et al, 2004^45^	**Design:** RCT of three surgical techniques **Procedure:** (A) PLTR (Kettesy) (B) Tarsal advance and rotation variant (C) Tarsal grooving variant **Location:** India **Number of patients:** 77 **Number of eyelids:** 90 **Case severity:** moderate/severe entropion **Surgeon grade:** ophthalmologist **Follow-up period:** 6 months	**Follow-up rates:** 96.7% (87/90) **Recurrence rates:** (A) 0% (0/28) (B) 6.9% (2/29) (C) 3.3% (1/30) **Associations with recurrence:** none recorded **Complications:** Lid notch (A) 30.0% (9/30) (B) 20.0% (6/30) (C) 33.3% (10/30)	Procedures (A) No dissection between ant. and post. lamellae of proximal portion in PLTR. Sutures emerge inferior to lash line. (B) Ant. and post. lamellae dissected via post. tarsotomy. No gray line incision. (C) Includes dissection of the ant. lamella is dissected from the tarsal plate
	**Outcome measure:** “anatomical correction of entropion (no contact of lashes with the globe in primary gaze)”	Granuloma (A) 10.0% (3/30) (B) 16.7% (5/30) (C) 10.0% (3/30) Irregular contour (A) 10.0% (3/30) (B) 3.3% (1/30) (C) 3.3% (1/30) Localised madarosis (C) 3/3% (3/30)	No significant difference found between outcomes of the three procedures. However, the sample size is small.

BLTR = bilamellar tarsal rotation; c. = approximately; F/U = follow-up; IECW = integrated eye-care worker; PLTR = posterior lamellar tarsal rotation; RCT = randomized controlled trial; TA&R = tarsal advance and rotation; TF = trachoma follicular; TI = trachoma inflammatory; TTC = tetracycline.

a Both studies derived from same data set.

**Table 4:** Barriers to Surgery

Authors	Study Description	Surgical Uptake	Barriers
Courtright, 1994^40^	**Design:** Prospective cohort **Location:** Malawi **Number of participants:** 29 **Follow up period:** 9-12 months	37.9%	• Far distance from main road • Not knowing another woman who had received surgery • Unilateral TT • Not widowed

West et al, 1994 (1)^136^; Oliva et al, 1997 (2)^98,a^	**Design:** Prospective cohort **Location:** Tanzania **Number of participants:** 200 **Follow up period:** (1) 2 years and (2) 7 years	18% at 2 years 27.4% at 7 years	• Lack of symptoms • Lack of time • Additional costs • Lack of escort • Children at home • Transport difficulties • Lack of money • Don't want surgery • Poor knowledge about service • Clinic failures (patient attended)

Bowman et al, 2000^24^	**Design:** Paired cluster randomized trial of (A) Health centre based surgery (B) Village based surgery **Location:** Gambia **Number of participants:** 158 **Follow up period:** one year	(A) 44% (B) 66% (RR, 1.49; 95% CI, 1.11–2.01; p = 0.009)	• Cost • Distance

Rabiu and Abiose, 2001^102^	**Design:** Cross-sectional **Location:** Nigeria **Number of participants:** 101 **Follow up period:** cross-sectional study	90% (of people with TT had not sought treatment)	• Cost • Lack of symptoms • Distance • Lack of escort

Bowman et al, 2002^22^	**Design:** Prospective cohort **Location:** Gambia **Number of participants:** 148 **Follow up period:** 12 months	23%	• Mild symptoms • Previous bad surgical experience • Fear • Happy to epilate • Using traditional eye medicines • Family opposition • Too expensive • Lack of time • Lack of escort • Do not know how to access surgery • Seasonal income • Geographic • Heard radio broadcast
Mahande et al, 2007^87^	**Design:** Prospective cohort of villages with trachoma education provide by: (A) Village leaders (B) School teachers **Location:** Tanzania **Number of participants:** 225 **Follow up period:** 1 year	44.8% overall (A) 52.1% (B) 36.5% in villages served by school teacher education (RR, 1.4; 95% CI, 0.9–2.1; p = 0.006)	• Unilateral TT • Surgical provision failure • Less effective TT surgery education program • Geographic • Clinic failures (patient attended)

Habte et al, 2008^61a^	**Design:** Case-Control **Location:** Ethiopia **Number of participants:** 135 cases, 141 controls **Follow up period:** n/a	n/a	• Burden of household chores • Indirect costs of surgery • Fear of surgery • Concerns about surgical outcome • Mild symptoms

95% CI = 95% confidence interval; RR = rate ratio; TT = trachomatous trichiasis.

a Same study population in both studies.

## Outline

I. Introduction
II. Global distribution and burden
III. Pathophysiology and natural history of trachomatous trichiasis
IV. Clinical assessment
  A. Lid and lash position
  B. Tarsal conjunctiva
  C. Cornea
  D. Differential diagnosis
V. Anatomy of the upper eyelid
VI. A historical perspective on trichiasis treatment
VII. Treatments for trichiasis and entropion
  A. Lash treatments
    1. Epilation
    2. Lash follicle destruction procedures
      a. Cryotherapy
      b. Electrolysis
      c. Laser
      d. Radiotherapy
    3. Surgical excision of aberrant lash-bearing follicles or tissue
  B. Corrective lid surgery
    1. Tarsal rotation procedures
      a. Bilamellar tarsal rotation
      b. Posterior lamellar tarsal rotation
    2. Tarsal advance procedures
      a. Tarsal advance
      b. Tarsal advance and rotation
    3. Posterior lamellar lengthening procedures
    4. Anterior lamellar and lid margin procedures
      a. Anterior lamellar repositioning
      b. Eversion splinting
      c. Tarsal grooving (tarsal wedge resection/ Cuenod-Nataf / anterior tarsotomy)
    5. Tarsectomy
VIII. Outcomes of trachomatous trichiasis treatment
  A. Trichiasis recurrence
    1. Surgery and surgeon-related factors
      a. Surgical procedure
      b. The surgeon
      c. Other factors
    2. Patient-specific factors
      a. Pre-operative disease severity and recurrent disease
      b. Infection
      c. Other factors
  B. Visual acuity and corneal opacity
  C. Surgical complications
    1. Stitch granulomas
    2. Wound infection
    3. Lid notching and overcorrection
    4. Other complications
IX. Treatment of trachomatous trichiasis in endemic settings
  A. Choice of operation
  B. Indications for surgery
  C. Delivering TT surgery services in endemic countries
    1. Who should operate?
    2. How should the service be structured?
    3. Barriers to surgery
    4. The cost of treatment
X. Conclusion
XI. Method of literature search
